# Influences of water chemical property on infiltration into mixed soil consisting of feldspathic sandstone and aeolian sandy soil

**DOI:** 10.1038/s41598-020-76548-7

**Published:** 2020-11-11

**Authors:** Ruiqing Zhang, Zenghui Sun, Gang Li, Huanyuan Wang, Jie Cheng, Mingde Hao

**Affiliations:** 1 College of Resources and Environment, Northwest A&F University, Yangling, China; 2 Key Laboratory of Degraded and Unused Land Consolidation Engineering, The Ministry of Natural Resources, Xi’an, China; 3 Institute of Land Engineering and Technology, Shaanxi Provincial Land Engineering Construction Group Co., Ltd., Xi’an, China; 4 Shanxi Provincial Land Engineering Construction Group Co., Ltd, Xi’an, China

**Keywords:** Hydrogeology, Environmental impact

## Abstract

Water infiltration into the soil profile are related to the condition of the soil texture, soil bulk density, and water intensity, it is also affected by the physicochemical properties of the water. In this study, we tested the effect of two different chemical properties of water (groundwater for irrigation and naturally accumulated water) on water infiltration in seven different mixed soil consisting of different ratios of feldspathic sandstone and aeolian sandy soil (1:0, 5:1, 2:1, 1:1, 1:2, 1:5, 0:1) through laboratory soil column testing. Our results show that when the textures of the mixed soils are silty loam and sandy loam (ratios of feldspathic sandstone to aeolian sandy soil 1:0, 5:1, 2:1, 1:1 and 1:2), the infiltration time of the naturally accumulated water is significantly longer than the infiltration time of the groundwater for irrigation. When the mixed soil texture is loamy sand and sand (the ratio of feldspathic sandstone to sandy soil is 1:5 and 0:1), there was no significant difference in the infiltration time of the naturally accumulated water and of the groundwater for irrigation. Using water with the same chemical properties, the infiltration time in different ratios of mixed soil decreases from 1:0, 5:1, 2:1, 1:1, 1:2, 1:5, to 0:1. Using the same feldspathic sandstone to aeolian sandy soil ratio, the cumulative infiltration using naturally accumulated water is greater than that using groundwater for irrigation, and the difference in cumulative infiltration is greatest when the ratio of feldspathic sandstone to sandy soil is 2:1. The relationship between the cumulative infiltration and elapsed time is consistent with the Logarithmic model. The changes in wetting front migration distance are consistent with the changes in the cumulative infiltration. The infiltration characteristics of water in the mixed soil are affected by a combination of water chemical property and soil texture.

## Introduction

The Shanxi-Shaanxi-Inner Mongolia Energy Zone spans a total area of 5.44 × 10^4^ km^2^ and possesses an abundance of coal resources. It is a large coal mining area and a fragile ecological environment in China^1^. With large-scale surface open-pit mining, the original landforms and ecological environments have been seriously damaged. The soil structure in the area has also been seriously damaged and intense soil degradation and water erosion are present, rendering the ecosystem in the area even more fragile, and severely restricting the socio-economic development of the region. The soil types in this region are mainly aeolian sandy soil and loessial soil^2^. The structure of aeolian sandy soil is loose, and soil structure, as well as water and fertilizer retention are poor, making the soil prone to erosion. In addition, one-third of the area in the Shanxi-Shaanxi-Inner Mongolia Energy Zone has distributed regions of feldspathic sandstone^3^. Feldspathic sandstone has a low degree of diagenesis, weak cementation between the sand grains, and has poor structure. When it is dry, it is as hard as stone, but when it is wet, it becomes soft and muddy, and it is the main source of sediment in the Yellow River^4^. Because of the relatively strong hydrophilicity and adsorption of montmorillonite in the feldspathic sandstone, as well as the relatively poor water and fertilizer retention of aeolian sandy soil, the two types of soil are somewhat complementary in nature. The sand particle content of feldspathic sandstone is 19–30% and the finer particles make up 50–73% of the material by weight, while the sand particle content of sandy soil can be as high as 90%^5^. There are significant differences in the mechanical properties of the two types of soil. With an increasing ratio of feldspathic sandstone, the texture of mixed feldspathic sandstone and sandy soil can change from sandy soil to loamy sand, sandy loam, and silty loam^6^. In order to restore the productivity of the land in this region, some scholars have tried to mixed feldspathic sandstone and aeolian sandy soil in recent years and have achieved good results^7^.

Soil water infiltration is the process by which water flows downward into the soil and its storage and movement in the soil^8,9^. It is an important link in the water cycle that connects air water, surface water, soil water, and groundwater^10^. The study of soil water infiltration is not only helpful to the development of a basic theory of soil water infiltration and migration in unsaturated zones, but also helpful to the comprehensive evaluation of surface and groundwater resources, which provides a scientific basis to reasonably determine the technical parameters of farmland irrigation. Soil water infiltration in addition to the influence of initial water content, soil texture, and water flow intensity, is also affected by factors such as water quality. The physicochemical properties of the water can significantly affect the soil water potential and hydraulic conductivity. The type and content of solute in water will affect water density, surface tension, and viscosity coefficient, which will affect the movement of water in the soil^11^. The soil hydraulic conductivity is related to the composition and content of exchangeable cations in the soil solution and the concentration of soluble electrolytes. The hydraulic conductivity decreases with the increase in sodium adsorption ratio. An increase in sodium ions in irrigation water can lead to shrinkage of soil particles and dispersion and expansion of colloidal particles, which in turn can affect the permeability of the soil^12,13^. Soil water infiltration is mainly affected by soil mechanical composition, water-stable aggregates, soil bulk density, organic matter content, and initial soil moisture content^14–17^. The main properties of soil, such as mechanical composition, water-stable aggregate and soil bulk density will differ in mixed soils consisting of different ratios of feldspathic sandstone and aeolian sandy soil. A series of studies have shown that the combination of feldspathic sandstone and aeolian sandy soil has significant water-saving effects. The main reason for this is that the colloid in feldspathic sandstone can effectively enhance the surface hydrophilicity of sand particles and the pore water retention of aggregates, effectively retaining water in the mixed soil layer and reducing leakage to the deep layer; the surface soil layer dries and contracts easily and breaks away from the bottom soil layer, cutting off capillary water movement^18,19^. Therefore, our current study on the water infiltration characteristics of feldspathic sandstone and sand mixed soil with different ratios and as well as the mechanism of different water quality on the water infiltration of mixed soil is of great significance in determining the ideal water-saving soil composition and in promoting plant growth and ecological restoration in the sandy and arid areas of the world^20,21^. Although there are many studies on the factors that influence soil water infiltration^22–27^, most of these studies use single soil types. There are fewer studies of this type on new soils obtained by the combination of feldspathic sandstone and sand.

In this paper, feldspathic sandstone and aeolian sandy soil were mixed at seven different volume ratios. Groundwater for irrigation and naturally accumulated water were used to carry out laboratory soil column experiments to study the effect of water chemical property on the soil water infiltration process in mixed soil. Our aim is to recognize in depth the soil water infiltration process and to construct a model of soil water movement to provide a scientific basis and reference for field irrigation using water with different chemical properties.

## Results

### Influence of water chemical property on infiltration time of mixed soil

**Figure 1:**
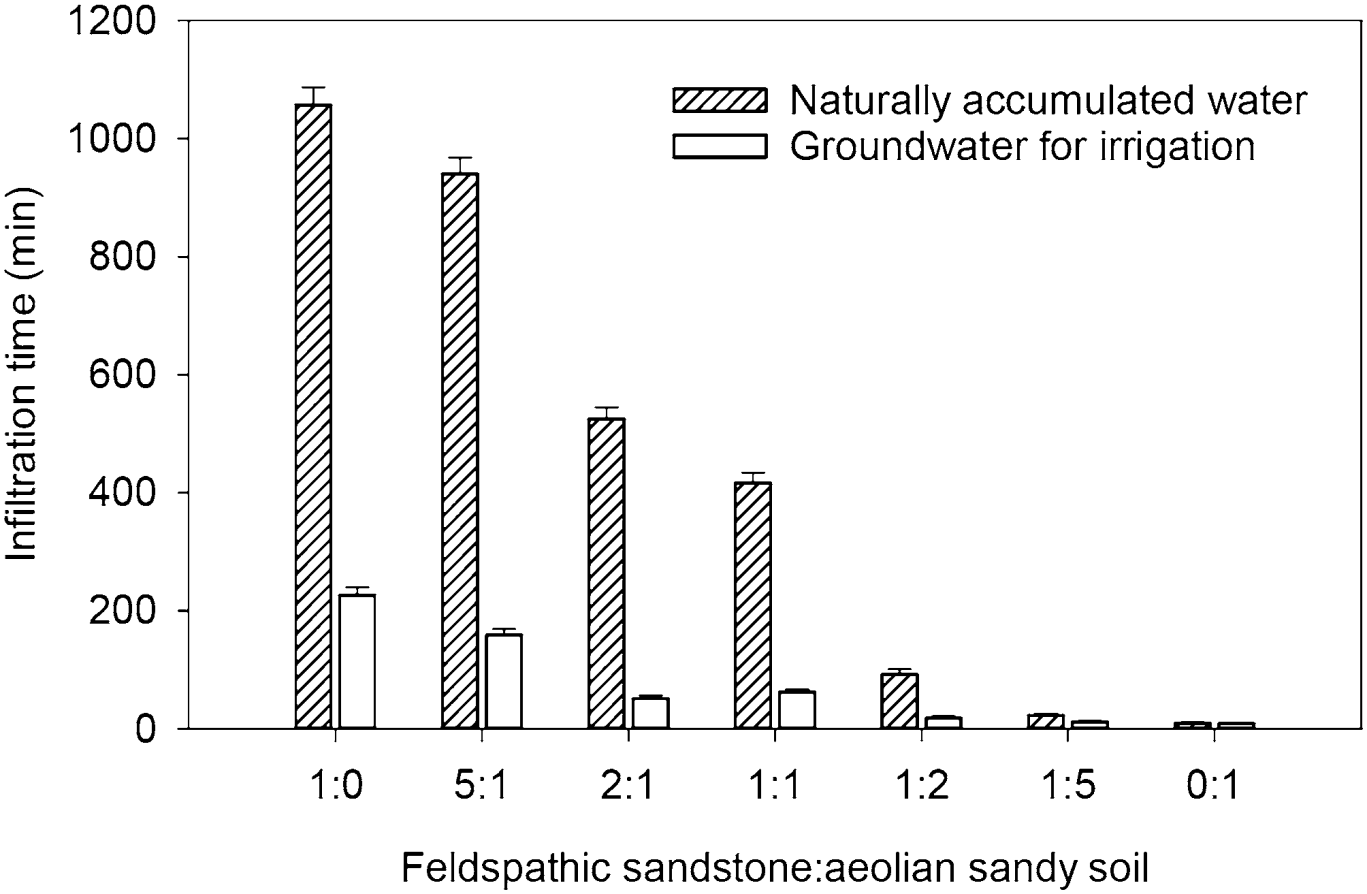
Infiltration time of water with different chemical properties in mixed soil (https://www.sigmaplot.co.uk/products/sigmaplot/produpdates/prod-updates18.php).

With increasing ratios of feldspathic sandstone in the mixed soil, the texture of the mixed soil changed from sand to loamy sand, sandy loam, and silty loam (Table 1). When the soil texture is silty loam and sandy loam (the ratio of feldspathic sandstone to aeolian sandy soil is 1:0, 5:1, 2:1, 1:1 and 1:2), the infiltration time of naturally accumulated water was significantly longer (*p* < 0.05) than that of the groundwater for irrigation (Fig. 1). The difference in infiltration time between the two water chemical properties was greatest at a ratio of 1:0 of feldspathic sandstone to aeolian sandy soil. When the mixed soil texture is the loamy sand and sand (the ratio of feldspathic sandstone to aeolian sandy soil is 1:5 and 0:1), there are no significant differences in the infiltration time between natural and groundwater for irrigation (*p* > 0.05). This indicates that soil water infiltration time is affected by the water chemical properties as well as the soil texture. Using naturally accumulated water, the infiltration time of the different ratios of mixed soil are 1:0 > 5:1 > 2:1 > 1:1 > 1:2 > 1:5 > 0:1. Using groundwater for irrigation, the infiltration time in the different ratios of mixed soil are slightly different, the infiltration time is 1:0 > 5:1 > 2:1 ≈ 1:1 > 1:2 > 1:5 ≈ 0:1.

**Table 1:** Composition and texture of mixed soil.

Mixing ratios	1:0	5:1	2:1	1:1	1:2	1:5	0:1
**Particulate composition (%)**							
Sand	34.85	42.44	50.37	52.65	60.7	83.33	95.31
Silt	58.09	51.52	44.40	42.26	35.08	14.97	4.45
Clay	7.06	6.04	5.23	5.09	4.22	1.7	0.24
**Texture**							
	Silty loam	Silty loam	Sandy loam	Sandy loam	Sandy loam	Loamy sand	Sand

### Effect of water chemical property on infiltration rate of mixed soil

**Figure 2:**
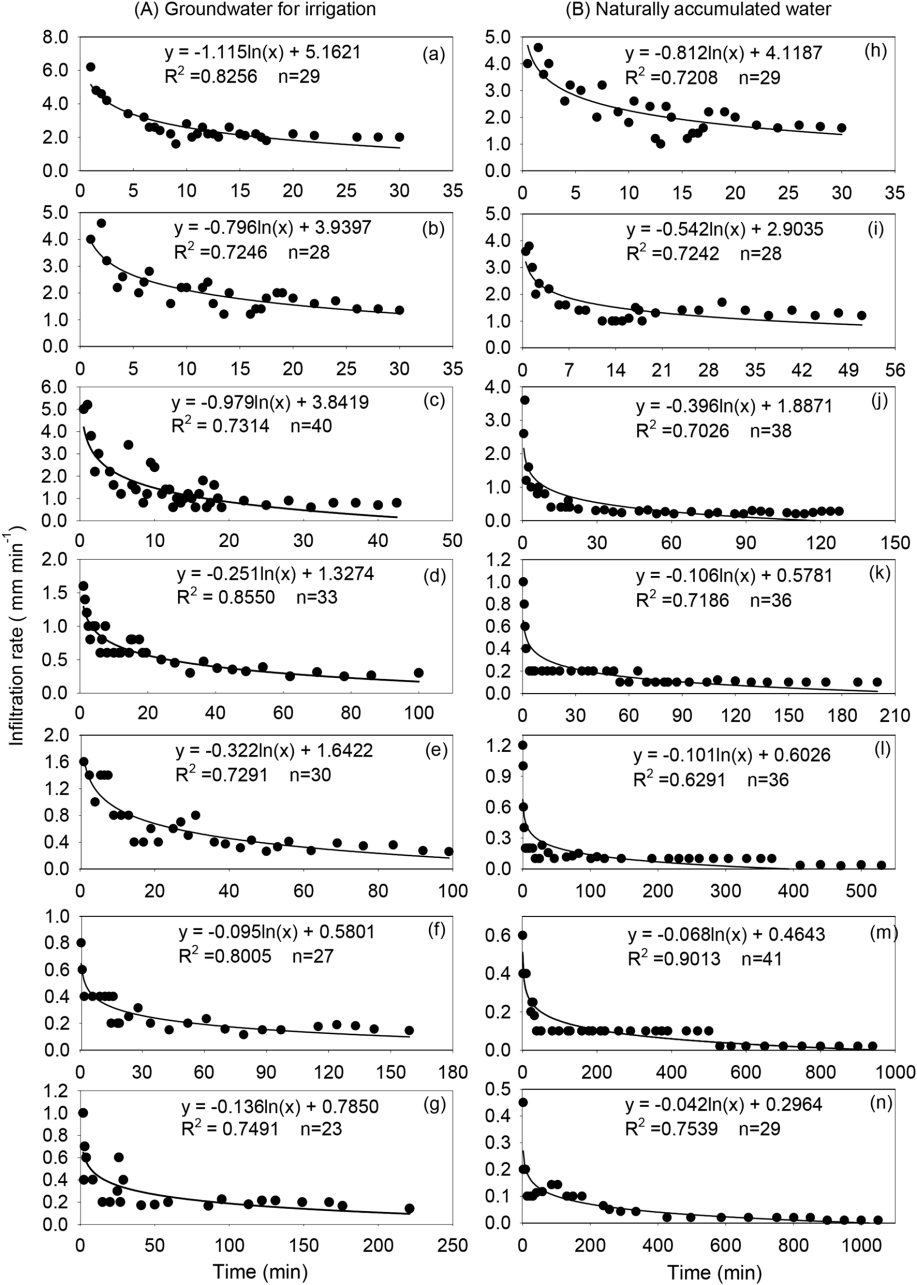
Changes of in infiltration rate with time for water with different chemical properties and mixed soilsof different water chemical properties in compounded soil. **a** and **h** represent 0:1, **b** and **I** represent 1:5, **c** and **j** represent 1:2, **d** and **k** represent 1:1, **e** and **i** represent 2:1, f and m represent 5:1, g and n represent 1:0 (https://www.sigmaplot.co.uk/products/sigmaplot/produpdates/prod-updates18.php).

**Figure 3:**
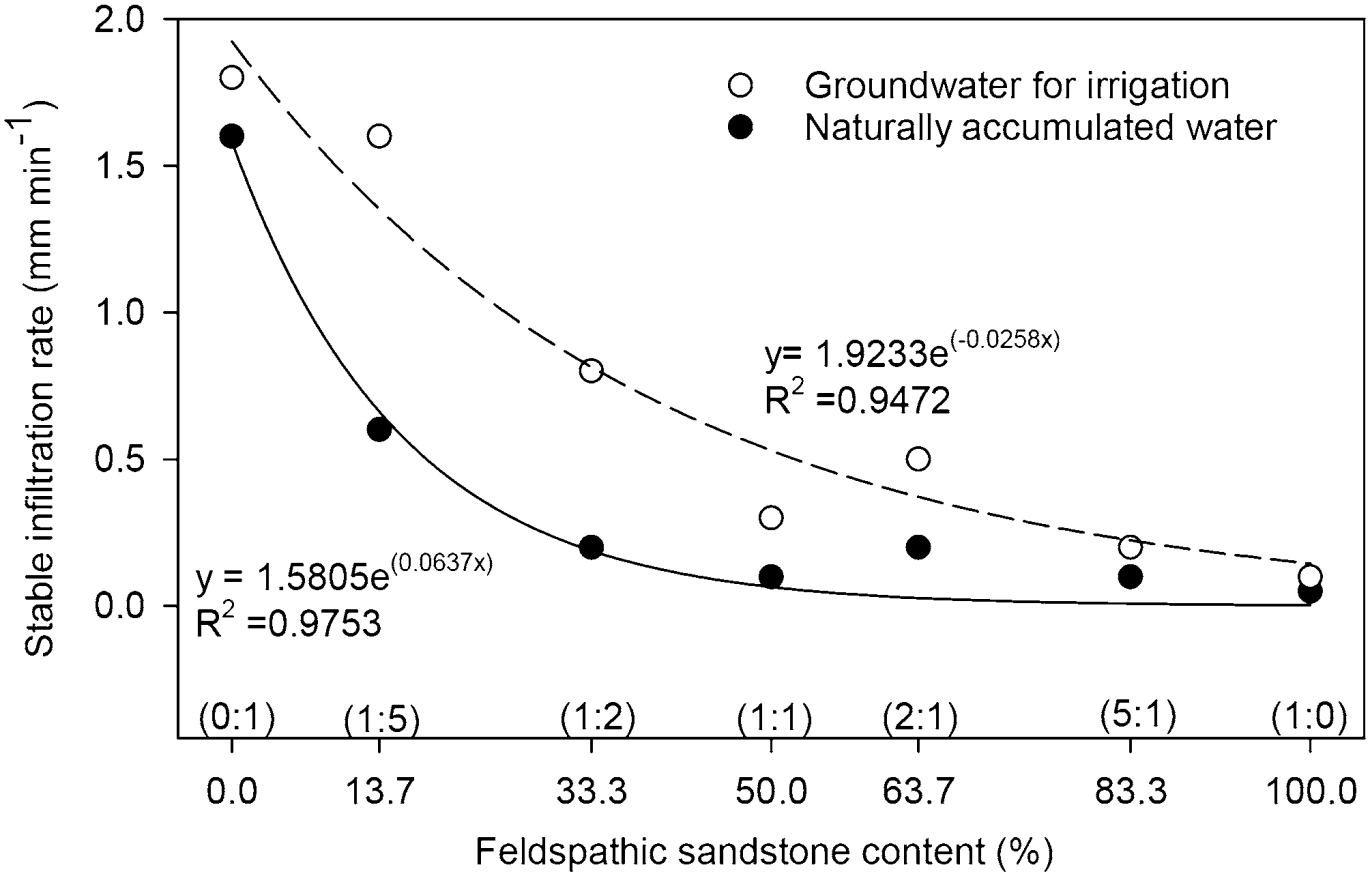
The stable infiltration rate vs. feldspathic sandstone content relationships for water with different chemical properties.Variation trend of stable infiltration rate with content of feldspathic sandstone under different water chemical properties (https://www.sigmaplot.co.uk/products/sigmaplot/produpdates/prod-updates18.php).

The different properties of water showed similar infiltration rates in the mixed soil and both showed a downward trending logarithmic function with time (Fig. 2). The best fit curve changes from steep to gentle within a short time from the start of soil infiltration and gradually becomes constant. In mixed soil containing the same ratio of feldspathic sandstone to aeolian sandy soil, the infiltration rate of naturally accumulated water is significantly lower than that of groundwater for irrigation. The infiltration rate of naturally accumulated water and groundwater for irrigation increases with the decrease of feldspathic sandstone content in the mixed soil. At the same time, the infiltration rate is the lowest in soil containing a ratio of feldspathic sandstone to aeolian sandy soil of 1:0, while it is highest in the 0:1 ratio soil. Using water with the same chemical properties, the infiltration rate in soil with different mixing ratios decreased with the increase of feldspathic sandstone content (Fig. 3). With increased infiltration time, the infiltration rate of mixed soil tends to be stable, but the stable infiltration rate was the smallest, using both naturally accumulated water and groundwater for irrigation, in mixed soil with a ratio of feldspathic sandstone to aeolian sandy soil of 1:0. The stable infiltration rates were 0.05 and 0.1 mm min^−1^, respectively. The stable infiltration rate was the highest when the ratio of feldspathic sandstone to aeolian sandy soil was 0:1, and the infiltration rates were 1.6 and 1.8 mm min^-1^ for naturally accumulated water and groundwater for irrigation, respectively.

### Influence of water chemical property on cumulative infiltration of mixed soil

**Figure 4:**
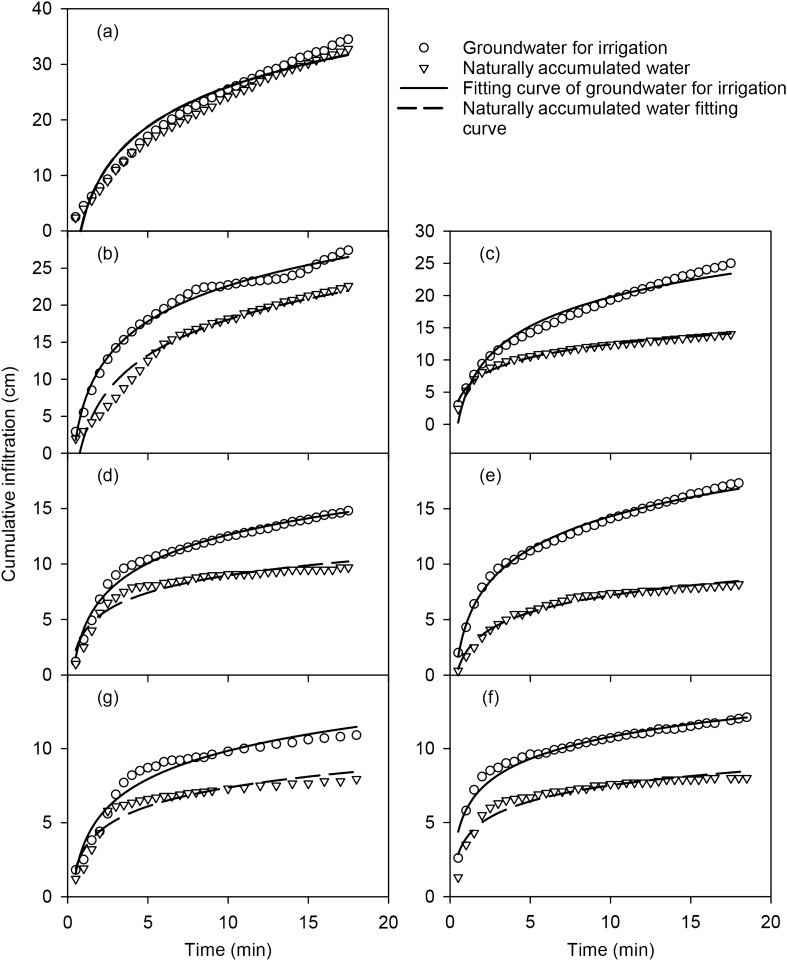
Changes in cumulative infiltration with time for water with different chemical properties and mixed soilsof water from different chemical properties in compounded soil. **a** represent 0:1, **b** represent 1:5, **c** represent 1:2, **d** represent 1:1, **e** represent 2:1, **f** represent 5:1, **g** represent 1:0 (https://www.sigmaplot.co.uk/products/sigmaplot/produpdates/prod-updates18.php).

There is a logarithmic relationship between the cumulative infiltration and changes in infiltration time of water from different chemical properties in mixed soil (Table 2). The coefficients of determination R^2^ are all above 0.903, and the fit of the cumulative infiltration of the groundwater for irrigation is better than that of the naturally accumulated water. During the initial stage of infiltration, the infiltration rate using the groundwater for irrigation is higher in the different mixed soil (Fig. 4), that is, the slope of the cumulative infiltration curve at the initial stage of infiltration is steeper, and the infiltration time is longer. The cumulative infiltration of different mixed soil is continually increasing. For mixed soil of the same rate, the cumulative infiltration of groundwater for irrigation is greater than that of natural water within the same time period (Fig. 4, Table 2). Using water with the same chemical properties and the same infiltration time, the cumulative infiltration of soil mixed with different ratios of feldspathic sandstone and aeolian sandy soil is 0:1 > 1:5 > 1:2 > 1:1 > 2:1 > 5:1 > 1:0.

**Table 2:** Fitting relationship between cumulative infiltration and time of mixed soil.

Mixing ratios	Water	Fitting equation	R^2^
0:1	Naturally accumulated water	y = 9.8746ln(x) + 2.0014	0.950
	Groundwater for irrigation	y = 10.331ln(x) + 2.1683	0.951
1:5	Naturally accumulated water	y = 6.9446ln(x) + 2.0723	0.962
	Groundwater for irrigation	y = 6.9421ln(x) + 6.64	0.988
1:2	Naturally accumulated water	y = 2.9645ln(x) + 5.608	0.972
	Groundwater for irrigation	y = 6.5086ln(x) + 4.7732	0.987
1:1	Naturally accumulated water	y = 2.2499ln(x) + 3.7995	0.924
	Groundwater for irrigation	y = 3.6896ln(x) + 4.1361	0.986
2:1	Naturally accumulated water	y = 2.219ln(x) + 2.0825	0.984
	Groundwater for irrigation	y = 4.2272ln(x) + 4.5766	0.993
5:1	Naturally accumulated water	y = 1.5523ln(x) + 3.9338	0.914
	Groundwater for irrigation	y = 2.1376ln(x) + 5.8598	0.957
1:0	Naturally accumulated water	y = 1.8259ln(x) + 3.1636	0.903
	Groundwater for irrigation	y = 2.7538ln(x) + 3.5134	0.952

### Effect of water chemical property on migration of wetting front of mixed soil

**Figure 5:**
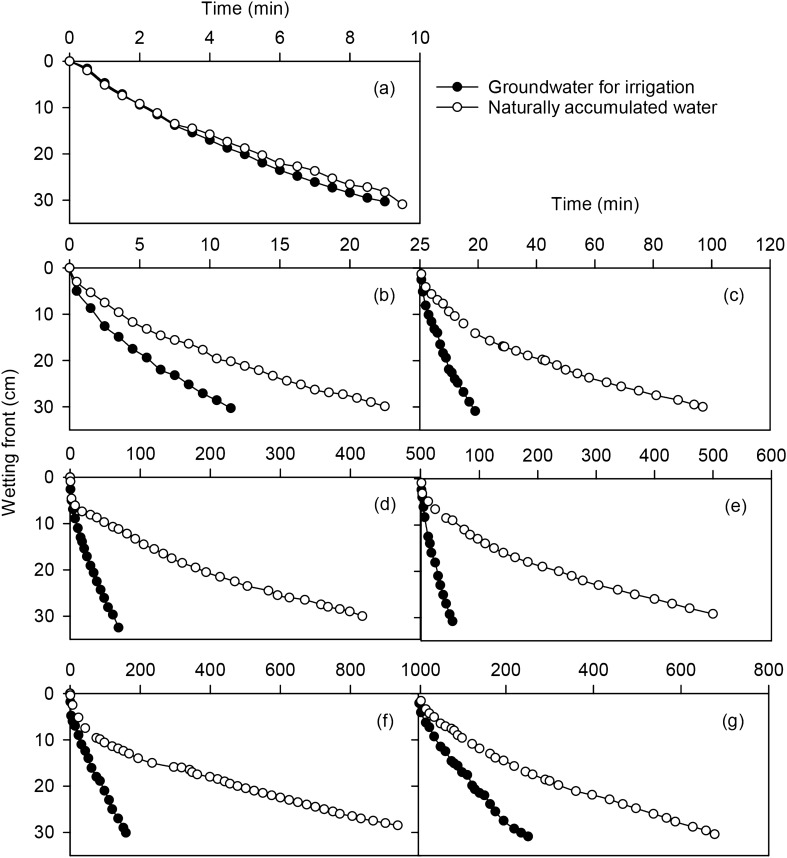
Effects of different water chemical properties on wetting front migration for various ratios of mixed soilsin various ratios of compounded soil. **a** represent 0:1, **b** represent 1:5, **c** represent 1:2, **d** represent 1:1, **e** represent 2:1, **f** represent 5:1, **g** represent 1:0 (https://www.sigmaplot.co.uk/products/sigmaplot/produpdates/prod-updates18.php).

The downward migration distance of water from different chemical properties increases with infiltration time, and the wetting front moves faster at the initial infiltration, but later the migration rate gradually decreases (Fig. 5). The migration pattern of the wetting front using water with different chemical properties in different mixed soil is similar, which shows that the wetting front movement is faster using groundwater for irrigation and slower using naturally accumulated water (Fig. 5). When the infiltration time is 9 min, the groundwater for irrigation in soil with mixing ratios of 1:0, 5:1, 2:1, 1:1, 1:2, 1:5, and 0:1 migrated 5.0, 6.2, 10.6, 10.1, 19.4, 26.0, and 30.3 cm, respectively, while the migration distances of the naturally accumulated water were 2.0, 2.7, 4.6, 6.4, 8.3, 17.0, and 28.3 cm, respectively. Decreasing the feldspathic sandstone content reduces the difference between the migration of the wetting front between the groundwater for irrigation and the natural water. Therefore, the influence of water chemical property on the wetting front migration distance of different mixed soil is also determined to some extent by the soil texture.

## Discussion

### Influence of water chemical property on infiltration time and infiltration rate in mixed soil

The process of soil infiltration is mainly affected by different water chemical properties and soil texture. The infiltration rate is the volume of water that has infiltrated the soil per unit time. The stable infiltration rate is the rate at which the soil infiltration rate has stabilized, which can reflect the infiltration capacity of the soil. Using water with the same chemical properties, the higher the feldspathic sandstone content, the slower the infiltration rate, mainly because as the content of the feldspathic sandstone in the mixed soil increases, the soil texture changes from sand to silt loam, and the soil bulk density and non-capillary porosity decrease, while the degree of porosity and capillary porosity increase, and the increase in soil clay content increases the fine pores in the soil, strengthening its ability to absorb water, and increases the water retention capacity, which leads to decreased soil water infiltration rate. This is consistent with findings reported by some researchers^28–32^.

Using water with different chemical properties, the sodium adsorption ratio of naturally accumulated water is significantly higher than that of groundwater for irrigation. The sodium ion content is higher in naturally accumulated water. After infiltrating the soil, the sodium ions in the water disperse soil aggregates, and the clay becomes more dispersed in the soil, leading to blocked pores and a reduction in soil porosity^33^. In addition, the high sodium adsorption rate in the naturally accumulated water can make sodium the dominant cation in the soil, so that part of the exchangeable calcium and magnesium are replaced by sodium, causing shrinkage of soil particles and dispersion and expansion of colloidal particles^34^, which can also lead to the reduction of soil pores^35^, affecting the permeability of the soil, thus affecting the soil water movement pathways, and ultimately the infiltration rate of water in the soil is reduced^36^. In this study, we found that using the same ratios of feldspathic sandstone and aeolian sandy soil in mixed soil, the infiltration rate of naturally accumulated water is significantly lower than that of groundwater for irrigation, which is consistent with Lubomír Lichner and other researchers’ conclusion that water chemical property is related to wettability^37–40^.

### Influence of water chemical property on cumulative infiltration in mixed soil

According to Darcy's law, the cumulative infiltration is affected by soil hydraulic conductivity and soil water potential gradient, and soil hydraulic conductivity is mainly determined by soil texture, bulk density, and structure^41^. In soil with the same mixing ratios of feldspathic sandstone and aeolian sandy soil, the cumulative infiltration of groundwater for irrigation is significantly greater than the cumulative infiltration of naturally accumulated water, which may be related to the conductivity of the infiltrating water. The conductivity of naturally accumulated water is significantly higher than that of groundwater for irrigation^42^. Some studies have shown that the increase of salt concentration in soil solution can promote soil water movement^43^, but it is not necessarily the case that the higher the salt concentration, the greater the cumulative infiltration. The conductivity of naturally accumulated water in this study is higher than that of the groundwater for irrigation^44^. In our study, the conductivity of the naturally accumulated water is six times that of the groundwater for irrigation. With longer infiltration time, the total amount of sodium ions entering the soil increases, the soil particles disperse and the clay swells, which damages the soil agglomerates to an extent, resulting in a slow reduction in the increase in soil infiltration.

In mixed soil Using water with the same chemical properties, cumulative infiltration decreases with the increase of the content of feldspathic sandstone. This may be due to the increase in soil cohesion, increasing water absorption and decreasing the infiltration rate of the soil^45^. On the other hand, the soil water retention capacity increases with the increase of soil salinity. In this study, the initial salt content of the mixed soil with a compound ratio of 1:0 feldspathic sandstone to aeolian sandy soil is significantly less than that of sandy soil with a ratio of 0:1. The destructive effect of sodium ions on the aggregate structure of 1:0 mixed soil is more obvious. Therefore, the decrease in infiltration capacity of 1:0 mixed soil is greater when using water with different chemical properties. When studying the water infiltration characteristics of different soil types in the desert steppe, Chen et al.^46^ also found that the aeolian sandy soil has loose structure, good permeability, the largest cumulative infiltration per unit time, high viscosity, firm structure, and high hardness. The hardness of the bedrock differentiated residual soil makes it difficult to infiltrate, and the cumulative infiltration is low. Barton et al.^47^ has shown that the cumulative infiltration of aeolian sandy soil is the highest, followed by loess and is lowest in feldspathic sandstone, which is consistent with the results of our study.

### Effect of water chemical property on migration of wetting front in mixed soil

Water chemical property has a significant impact on the wetting front movement in mixed soil. In this study, using the same water for infiltration, the infiltration rate of different mixed soil led to a difference in the wetting front migration distance. The time difference between the high ratios of feldspathic sandstone and the low ratios is large. When the ratio of feldspathic sandstone is high, the migration time of the wetting front is long, and the migration time of the wetting front of the feldspathic sandstone soil is 26.8 times that of aeolian sandy soil, indicating that feldspathic sandstone can effectively solve the problem of excessive leakage of aeolian sandy soil. Adding feldspathic sandstone to aeolian sandy soil can increase the retention time of water and improve the water retention capacity of aeolian sandy soil, which is consistent with previously published research results^48^.

Under the same mixing ratio, the wetting front migration speed of groundwater for irrigation is higher than that of naturally accumulated water. Studies have shown that the soil water conductivity and water holding capacity are determined by the conductivity of the infiltration water and the amount of sodium ions entering the soil solution. The infiltration water salt ions can enhance the soil water conductivity and water retention capacity to some extent^49^, but as the salt ion content increases, the soil infiltration capacity decreases. In this study, the conductivity and sodium adsorption ratio of naturally accumulated water is more than six times that of groundwater for irrigation. At this point, the destructive effect of increased sodium adsorption ratio on soil has exceeded the beneficial effect of salt ions, leading to a high percentage of soil exchangeable sodium, which makes the soil permeability worse and the wetting front slower^50^.

## Conclusions

1. Water chemical property has a significant effect on soil infiltration time and infiltration rate. In soil with the same quality, the soil water infiltration process in mixed soil with higher content of feldspathic sandstone is more clearly affected by water chemical property, infiltration of naturally accumulated water takes longer than that of groundwater for irrigation. However, in the 0:1 and 1:5 feldspathic sandstone to sand soil, the difference between natural and groundwater for irrigation is not clear. Therefore, water chemical property affects soil infiltration, but infiltration is also affected by the soil texture.
2. The effect of water chemical property on soil infiltration varies with soil texture. In soil with the same texture, the cumulative infiltration of groundwater for irrigation in the time period is higher than that of naturally accumulated water. Using water with the same chemical properties, the cumulative infiltration in the same time was greatest in the 0:1 feldspathic sandstone to aeolian sandy soil.
3. The wetting front movement is affected by water chemical property and is also affected by soil texture. When the wetting front migration distance is from 0 to 30 cm, the wetting front movement of groundwater for irrigation is faster than that of naturally accumulated water in all mixed soil. Increasing the feldspathic sandstone content increases the difference between the migration of the wetting front between the groundwater for irrigation and the natural water.

## Materials and methods

### Test soil

This study selected feldspathic sandstone and aeolian sandy soil from Xiaojihan Township (N38° 22′ 49.01″, E109° 37′ 50.69″) in Yuyang District, Yulin City, northern Shaanxi Province. The particle size distribution is shown in Table 3.

**Table 3:** Composition of feldspathic sandstone and aeolian sandy soil.

Sample	Particulate composition (%)			Texture type
	Clay (< 0.002 mm)	Silt ( ≥ 0.002~< 0.05 mm)	Sand ( ≥ 0.05~< 2 mm)	
Aeolian sandy soil	0.24	4.45	95.31	Sand
Feldspathic sandstone	7.06	58.09	34.85	Silty loam

The collected feldspathic sandstone and aeolian sandy soil were naturally air-dried, then ground through a 2 mm sieve. The feldspathic sandstone (F) and the aeolian sandy soil (S) were thoroughly mixed in different mass ratios (m(F):m(S) = 0:1, 1:5, 1:2, 1:1, 2:1, 5:1, and 1:0). After mixing, the particle composition of the soil at different mass ratios was determined using a MS-200 Malvern laser particle size analyzer (Table 1).

### Basic properties of water used for water infiltration experiments

Soil infiltration experiments were carried out with groundwater for irrigation and naturally accumulated water to reveal the effect of water chemical property on soil infiltration in mixed soil containing different feldspathic sandstone and aeolian sandy soil ratios. Groundwater for irrigation is used for drinking water for residents in Xi'an, while naturally accumulated lake water was taken from an artificial lake at Fuping Research Base. The basic properties of the two types of water used in this experiment are shown in Table 4. The water pH values were measured with pH meter (Seven Easy Mettler Toledo, China). The electric conductivity values in groundwater for irrigation and naturally accumulated water were detected by conductivity meter. The sodium adsorption ratio (SAR) is the ratio of the Na^+^ concentration in solution to the square root of the average Ca^2+^ and Mg^2+^ concentration. The calculation formula is as follows:

1 $$SAR = \frac{{Na^{ + } }}{{\sqrt {\frac{1}{2}\left( {Ca^{2 + } + Mg^{2 + } } \right)} }}$$

**Table 4:** The chemical properties of various water.

Water	Sodium absorption ratio	pH	Electrical conductivity (µs cm^−1^)
Groundwater for irrigation	3.69	8.05	165.8
Naturally accumulated water	23.84	6.75	1126

where the Na^+^, Ca^2+^ and Mg^2+^ are all the ionic concentrations expressed by meq/L.

### Water infiltration in mixed soil

One-dimensional vertical infiltration experiments were carried out in the laboratory with mixed soil containing different ratios of feldspathic sandstone and aeolian sandy soil. In order to reduce error, this experiment used a small plexiglass column with a diameter of 5 cm and a height of 35 cm to carry out the soil infiltration experiment using a thin layer of water. All treatments used homogeneous soil columns with a height of 30 cm. According to the designed bulk density (1.4 g cm^3^), the soil tank was stratified every 5 cm, tamped and layered to prevent any influence on soil infiltration, and the soil moisture content was allowed to evenly distribute over the course of 24 h. In addition, filter paper was placed on the of the soil column to facilitate uniform infiltration. A glass ball was placed at the bottom of the soil column. During the experiment, water was supplied from a Markov bottle and a constant head of 3 cm water was used to test soil column infiltration. At the same time, the water level reading in the Markov bottle at different times was recorded, and the wetting front migration curve was drawn. The infiltration time is the time from the start of infiltration to the wetting front reaching a depth of 30 cm. Due to the short duration of the experiment, the effect of evaporation on the soil infiltration process was ignored.

During the experiment, readings on the Martens flask were taken to calculate the water infiltration volume and to determine the position of the wetting front. The corresponding infiltration time was taken as well. When the first reading is taken, the farthest point of the wetted zone visible around the cylindrical wall of the experimental column was selected as the wet front migration distance at that time. Subsequent wetting front distances are fixed in the direction of the cylinder wall and are measured at the farthest position of the visible wetted zone.

During the experiment, the level on the Markov bottle was recorded for each time period, which was used to calculate the cumulative infiltration volume and cumulative infiltration rate, reflecting the characteristics of soil infiltration process and infiltration performance. The level in the Markov bottle was recorded every 1 min for the first 20 min, and the change in Markov bottle level was recorded every 5 min after 20 min until the end of the experiment.

### Statistical analyses

SigmaPlot12.5 software was used for data collation and mapping. SPSS22.0 software was used to perform one-way ANOVA and regression analysis on the test data, and the least significant difference method (LSD method) was used for multiple comparisons. The significance level was *p* < 0.05, and the extremely significant level was *p* < 0.01.
